# Coordinated Parameter Identification Technique for the Inertial Parameters of Non-Cooperative Target

**DOI:** 10.1371/journal.pone.0153604

**Published:** 2016-04-26

**Authors:** Xin Ning, Teng Zhang, Yaofa Wu, Pihui Zhang, Jiawei Zhang, Shuai Li, Xiaokui Yue, Jianping Yuan

**Affiliations:** 1 College of Astronautics, Northwestern Polytechnical University, Xi’an, Shaan xi, People’s Republic of China; 2 Department of Mechanical & Manufacturing Engineering, Schulich School of Engineering, University of Calgary, Calgary, Alberta, Canada; Kyushu University, JAPAN

## Abstract

Space operations will be the main space missions in the future. This paper focuses on the precise operations for non-cooperative target, and researches of coordinated parameter identification (CPI) which allows the motion of multi-joints. The contents of this paper are organized: (1) Summarize the inertial parameters identification techniques which have been conducted now, and the technique based on momentum conservation is selected for reliability and realizability; (2) Elaborate the basic principles and primary algorithm of coordinated parameter identification, and analyze some special problems in calculation (3) Numerical simulation of coordinated identification technique by an case study on non-cooperative target of spacecraft mounting dual-arm with six joints is done. The results show that the coordinated parameter identification technique could get all the inertial parameters of the target in 3D by one-time identification, and does not need special configuration or driven joints, moreover the results are highly precise and save much more time than traditional ones.

## Introduction

Decentralized control is an effective strategy for interconnected large-scale systems. The important reason is that the high order system is decoupled into several ones. For space missions, such as on-orbit servicing and maintenance, the inertial parameters of the target are usually unknown (non-cooperative target). Even for the cooperative target, its inertial parameters will change due to the location error of capture point and its fuel consumption. However, if the inertial parameter, an important factor that affects the performance of control system, is not obtained accurately, it will not be able to guarantee the high precision control of space assembly formed by active and target spacecraft. Therefore, in order to ensure the control performance of two aspects, one is the relative position and attitude of the target and base, the other is the attitude and orbit of the assembly system, the on-orbit autonomous parameter identification must be performed to determine the inertial parameters of unknown targets, including the mass of the target, position vector of the target centroid with respect to the capture point and inertial moment of the target with respect to its centroid[1].

There are many different kinds of parameter identification techniques at present, in which techniques based on dynamics needs to measure angular acceleration of the joints, but it is difficult to measure angular acceleration and the sensor is inaccurate for space system[2] [3]; the multi-axis wrist force sensor based identification methods are generally used for ground robots, and the sensor signal is often accompanied by a large noise, which easily affects the accuracy[4]; the technique based on neural network is still in research stage and has not been practically applied to project yet[5]; adaptive control based parameter identification technique also has some disadvantages in terms of its realizability in engineering and dynamic characteristics of the controller[6, 7].

For the space multibody system, it is considered that the system is in the conservation of linear and angular momentum when the reaction engine jet does not work (i.e., the orbital dynamics are neglected). The parameter identification technique based on linear and angular momentum conservation was first proposed by Murotsu[8], and it was verified by the flight test of ETS-VII[9]. But Murotsu simplifies the system into a two-body model to analyze, making this method have some shortcomings, for example, (1)the model is based on the assumption of plane, so single identification can only obtain in-plane inertia parameters, while in 3D space, only implementation of repeatedly identification can we obtain the whole parameters of the target; (2)one-time identification can only contain a single joint motion, which is not convenient to combine with other theories; (3)the system is a single-manipultor model, so its configuration is limited. On the basis of the original method, the scholars have combined it with other theories, and expand to the model of the multi-hinge single-manipulator model in the two-dimensional plane. Through combining the recursive adaptive controller with parameter identification, Sharf planned the motion of joints which would cause minimal disturbance to base attitude during parameter identification[10].

This paper aims to propose a fast and accurate coordinated parameter identification technique for space non-cooperative target, in which only one-time identification can we obtain multi-hinge 3D motion data at two time points in the system dynamic response (in other words, it can be a valid identification). With the combination of angular and linear momentum conservation and the recursive kinematic equations, the technique requires the centroid position and velocity of each component except the target and attitude angle and angular velocity of all components (including the target) to solve the identification equation. The inputs required include the centroid position, velocity and attitude angle and angular velocity of the base (the angle and angular velocity of each hinge). Combined with the recursive kinematic equations, we can obtain the correlated input for identification equations. Through the solution of identification equations, all the inertial parameters of non-cooperative target can be identified (including mass, the position vector of the center of mass relative to the capture point and moment of inertia of the target). Finally, taking the assembly of active spacecraft with two 6-DOF manipulators and non-cooperative target as an example, the numerical simulation for the proposed identification technique is performed. Then through putting identified parameters of target into dynamical model, the absolute error of the effects of the estimated and theoretical inertial parameters of the target on the attitude and position of spacecraft base is obtained. This method can provide an effective way for the high precision control of assembly to identify the inertial parameters quickly and autonomously.

## Methods

### Coordinated Parameter Identification

**Fig 1:**
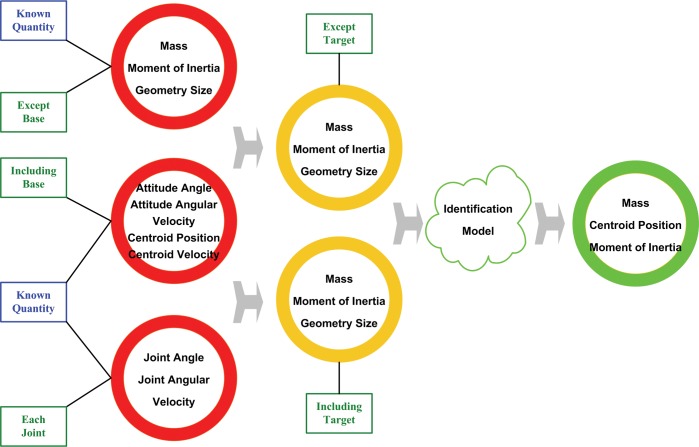
Process of coordinated identification technique.

As illustrated in the Fig 1, the inputs for coordinated parameter identification include the centroid position and the linear velocity of each component except the target as well as attitude angle and angular velocity of all components (including the target). Through the study of recursive kinematic equations, we know that only kinematic parameters of the base, coordinates and their derivations of joints are needed to obtain all relevant kinematic parameters of each component. In the end, these parameters are brought into the coordinated identification model to obtain mass, centroid position and moment of inertia of the target in 3D.

### Coordinated Identification technique

There are some problems for the original identification, such as a limitation on motion of joints, only applicable to single-manipulator system, the singularity of identification matrix, etc. Therefore, on the basis of the original techniques, the identification equation is improved, and the identification technique with motion of multiple joints is proposed. This technique is not bounded to the configuration of system and is suitable for solving 3D problems. All the unknown inertial parameters of the target can be totally obtained by one-time identification, besides; the motion of each joint can be conducted without limitation. In addition, there is no singularity for the identification matrix in the case of 3D motion.

**Fig 2:**
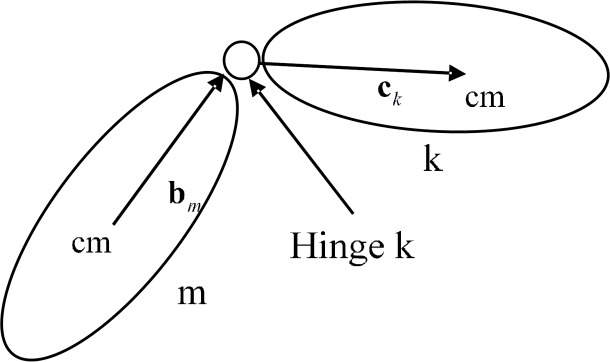
Inscribed object.

In order to describe the recursive kinematical equation, the inscribed object is introduced, as shown in the Fig 2.

The object marked as *m* is the inscribed object of the object marked as *k* and the joint between two objects is marked as *k*.

If the centroid position, linear and angular velocity of m-th object are known, the corresponding kinematic parameters of k-th object are defined as follows:

$$\omega_{k}=\omega_{m}+H_{k}^{\Omega }{\dot{q}}_{k}$$ (1)

$$r_{k}=r_{m}+b_{m}+c_{k}$$ (2)

$$v_{k}=v_{m}+\omega_{m}\times b_{m}+\omega_{k}\times c_{k}$$ (3)

Where, $H_{k}^{\Omega }$ is the unit direction vector of the k-th joint in inertial frame, it is related to the characteristics of the joint and attitude of m-th object, ${\dot{q}}_{k}$ is the first-order derivative of generalized coordinate *q*_*k*_, $H_{k}^{\Omega }{\dot{q}}_{k}$ is the relative angular velocity between two objects.

On the basis of the recursive kinematic equations, the coordinated parameter identification algorithm based on the conservation of linear and angular momentum is derived. In derivation, the target is marked as *n* for analyzing, and the centroid position of the base at the beginning is defined as the origin of inertial frame.

For the whole system, linear momentum **P** in inertial frame is expressed as follows:

$$P=\sum_{i=1}^{n}m_{i}v_{i}$$ (4)

Assuming that the initial linear momentum of system is zero, the velocity of each component should satisfy the equation as:

$$\sum_{i=0}^{n-1}m_{i}v_{i}+m_{n}v_{n}=0$$ (5)

Then the relation between kinematic parameters of the target and its inscribed object is brought into the above equation, and Eq 5 can be rewritten as

$$\frac{1}{m_{n}}\sum_{i=0}^{n-1}m_{i}v_{i}+v_{n_{i}}+\omega_{n_{i}}\times b_{n_{i}}+\omega_{n}\times c_{n}=0$$ (6)

The following equation can be obtained by linearizing Eq 6

$$\left[\sum_{i=0}^{n-1}m_{i}v_{i}\;{\tilde{\omega }}_{n}\right]\left[\begin{array}{c} \frac{1}{m_{n}}\\ c_{n} \end{array}\right]=-\left(v_{n_{i}}+{\tilde{\omega }}_{n_{i}}b_{n_{i}}\right)$$ (7)

The anti-symmetric matrix form of the vector $v={\left(\begin{array}{ccc} v_{1} & v_{2} & v_{3} \end{array}\right)}^{T}$ is defined as

$$\tilde{v}=\left[\begin{array}{ccc} 0 & -v_{3} & v_{2}\\ v_{3} & 0 & -v_{1}\\ -v_{2} & v_{1} & 0 \end{array}\right]$$ (8)

The angular momentum of the system in inertial frame is defined as:

$$L=\sum_{i=0}^{n-1}I_{i}\omega_{i}+m_{i}r_{i}\times v_{i}+I_{n}\omega_{n}+m_{n}r_{n}\times v_{n}$$ (9)

Based on the assumption that initial angular momentum of the system is zero, Eq 9 can be linearized similarly as the linear momentum conservation equation, thus the above equation can be expressed as

[∑i=1n−1miv˜i[#ωn]][cn'[I'#]]=−∑i=1n−1(Iiωi−miv˜i(ri−dno)) (10)

Where **d**_*no*_ denotes the centroid position vector of the n-th joint in inertial frame, the vector $v={\left(\begin{array}{ccc} v_{1} & v_{2} & v_{3} \end{array}\right)}^{T}$ is taken as an example to explain the matrix expression [#•], shown as below:

$$\left[\#v\right]=\left[\begin{array}{cccccc} v_{1} & v_{2} & v_{3} & 0 & 0 & 0\\ 0 & v_{1} & 0 & v_{2} & v_{3} & 0\\ 0 & 0 & v_{1} & 0 & v_{2} & v_{3} \end{array}\right]$$ (11)

While the concrete expression of the vector expression [#•] can be expressed as follows:

$$\left[I\#\right]={\left(I_{11},I_{12},I_{13},I_{22},I_{23},I_{33}\right)}^{T}$$ (12)

$$I=\left[\begin{array}{ccc} I_{11} & I_{12} & I_{13}\\ I_{12} & I_{22} & I_{23}\\ I_{13} & I_{23} & I_{33} \end{array}\right]$$ (13)

Where **I** is the symmetric matrix, and the inertia moment of the target is relative to its own centroid in inertial frame.

By combining the linear and angular momentum conservation equation, the corresponding identification equation can be obtained as:

[∑i=1n−1miviω˜n03×603×1∑i=1n−1miv˜i[#ωn]][1mncn'[I'#]]=[−(vni+ω˜nibni)−∑i=1n−1(Iiωi−miv˜i(ri−dno))] (14)

For the target, its centroid position relative to the capture point and its moment of inertia in inertial frame is closely related to the attitude of the body; their projection relation can be defined as

cn'=Ancn (15)

In'=AnInAnT (16)

They would change due to the rotation of target. In order to solve this, it is necessary to deal with the original model. The constraint that direction cosine matrix should satisfy is introduced, that is, its inverse matrix is the same as its transpose matrix, as shown below.

$$A^{T}A=A^{-1}A=I$$ (17)

The change of the centroid position of the target relative to the capture point and moment of inertia, which is caused by attitude of the target, is brought into the original model. Then Eq 14 could be written as:

$$\left[\begin{array}{ccc} \sum_{i=1}^{n-1}m_{i}v_{i} & {\tilde{\omega }}_{n}A_{n} & 0_{3\times 6}\\ 0_{3\times 1} & A_{n}{}^{T}\left(\sum_{i=1}^{n-1}m_{i}{\tilde{v}}_{i}\right)A_{n} & \left[\#A_{n}{}^{T}\omega_{n}\right] \end{array}\right]\left[\begin{array}{c} \frac{1}{m_{n}}\\ c_{n}\\ \left[I\#\right] \end{array}\right]=\left[\begin{array}{c} -\left(v_{n_{i}}+{\tilde{\omega }}_{n_{i}}b_{n_{i}}\right)\\ -A_{n}{}^{T}\sum_{i=1}^{n-1}\left(I_{i}\omega_{i}-m_{i}{\tilde{v}}_{i}(r_{i}-d_{no})\right) \end{array}\right]$$ (18)

After the linearization, the dynamic parameters needed to be solved are described in body-fixed frame of the target and they are constant in each state, which is convenient to process and analyze the subsequent data.

The kinematic parameters of each component, which are obtained from the system dynamics model established by the previous recursive method, are brought into the identification model, and then all the inertial parameters can be calculated through the least square method.

## Result and Discussion

### Simulation object

**Fig 3:**
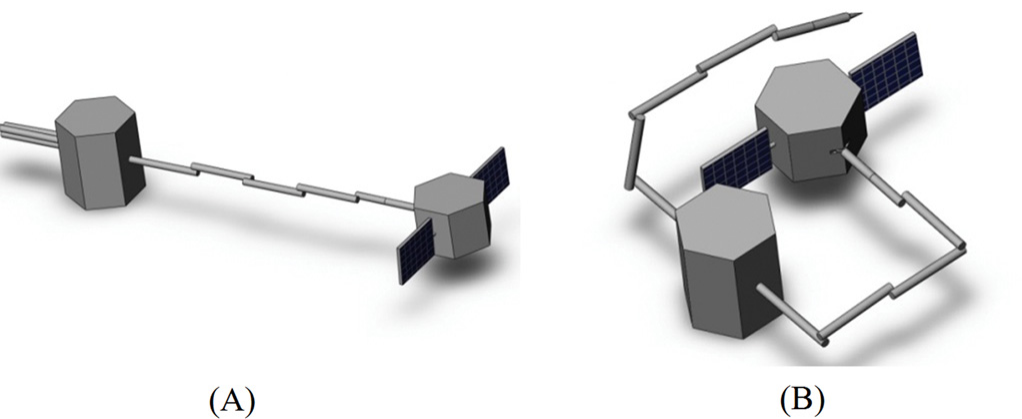
The configuration of system of CPI. (A) The initial configuration. (B) The final configuration.

The space platform with two 6-DOF manipulators is simulated. After the platform successfully captures the target, the relative position and attitude between the target and the base are adjusted by the motion of the capturing manipulator, while the operating arm is unfolded for subsequent operations. The system configuration in the initial time and the end time are shown in the Fig 3.

### Simulation process

**Fig 4:**
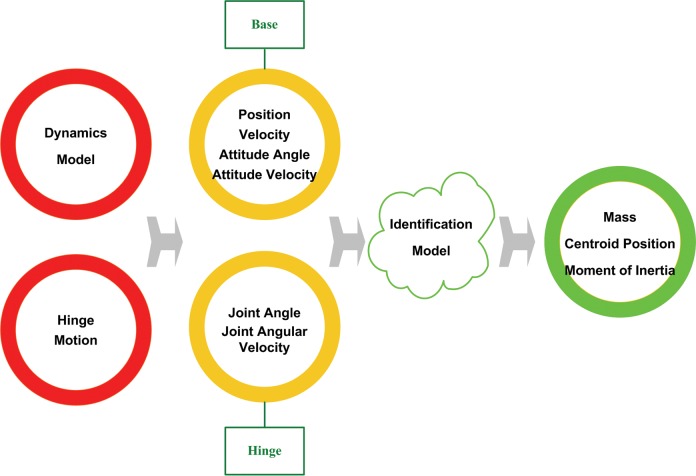
Process of CPI.

The flowchart of simulation is shown in the Fig 4. The dyanmics for multi-rigid body system is established through recursive method[11] and the motion planning of joint is performed, then by measuring system response, position, velocity, attitude angle and angular velocity of the base and also joint angle and angular velocity are obtained. At last, taking the above parameters as the identification model inputs, the whole unknown inertial parameters are obtained,

### Input condition of the dynamics model and the initial state of the system

**Fig 5:**
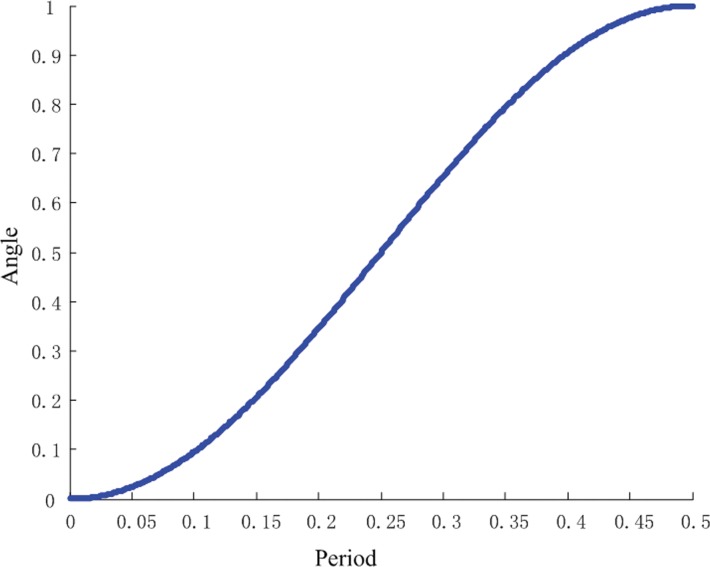
Angular of the driven joint.

The applied motion of the joint is a half cycle of cosine motion, and the change rate of the angle with time during half cycle is shown in the Fig 5. It can be seen from the Fig 5 that it is a cosine curve, and the corresponding angular velocity is zero at the initial time t = 0 and the terminal time t = T/2 in order to avoid impact caused by the sudden change of the joint angular velocity, which could make the changing process smoother.

### The system dynamic response

**Fig 6:**
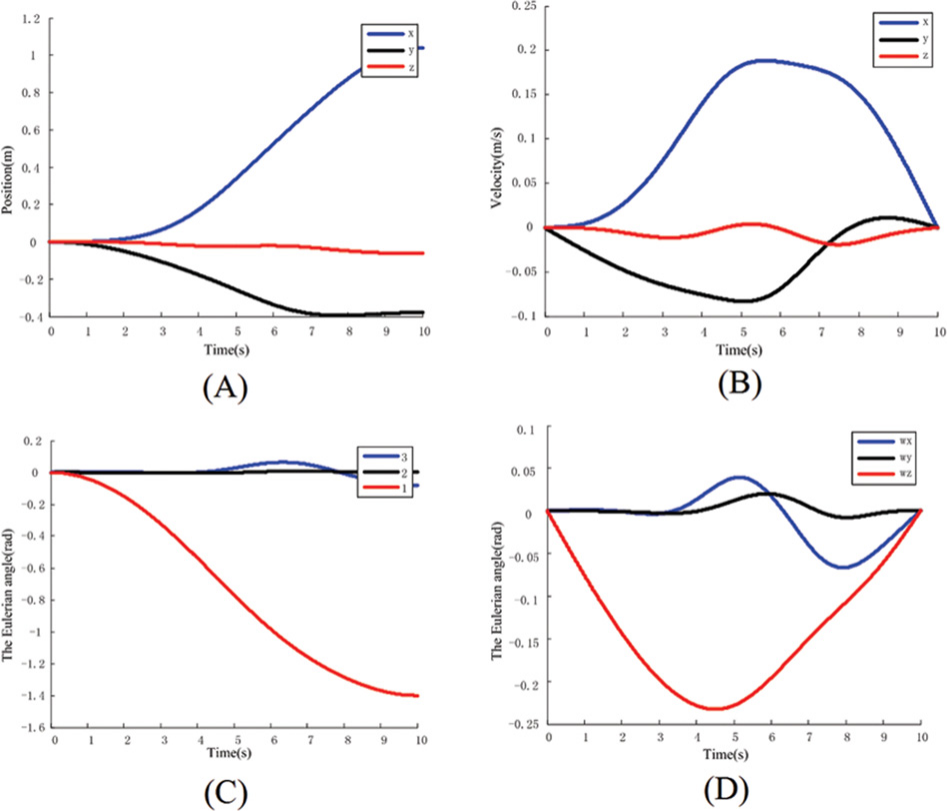
The base’s parameters in identification. (A) Position of Base. (B) Base’s velocity. (C) Position of Base. (D) Base’s velocity.

In the Fig 6, the dynamic response with time for the base is shown respectively. Simulation is built according to recursive method, and the system is assumed as a multi-rigid body system.

### Identification results and analysis

In simulation, at least two sets of data were required to identify the inertial parameters of target, and in order to increase the accuracy of the identification algorithm, the sampling period for the measurement data was set to 1s, the first time for sampling began from t = 1.0s (the system is static at the beginning, so it is useless to input the corresponding data to the identification model), so that ten sets of data from t = 1.0s to t = 10.0s could be collected, and then the ten sets of data were inputted into the model.

Finally, all the inertial parameters of the target were obtained by using the least squares method, and the results are shown in the Table 1.

**Table 1:** Results of CPI.

Inertial parameters	Theatrical value	Identified value	Relative error
m(kg)	100	99.897213	0.10%
c_x_(m)	0.4330127	0.434876	0.43%
c_y_(m)	0	0.001487	——
c_z_(m)	0	-0.000866	——
I_xx_(kgm^2^)	20	18.962848	5.19%
I_yy_(kgm^2^)	30	30.018296	0.06%
I_zz_(kgm^2^)	10	9.706598	2.93%
I_xy_(kgm^2^)	0	-0.279775	——
I_xz_(kgm^2^)	0	-0.09017	——
I_yz_(kgm^2^)	0	-0.075827	——

The identification result shows that there is a certain error exists. But the error term is not introduced in the dynamics model of the system, so the error may be caused by the truncation error of inputs. Thus, analysis of this phenomenon is performed. Selecting the digits after the decimal point as the independent variable to identify the quality of the target, the relationship between them was obtained, as shown in the Table 2.

**Table 2:** The identified mass from the data with different precision.

Digits after the decimal point	Identification results
1	234.521597
2	98.798019
3	99.015893
4	99.897745
5	99.895927
6	99.897213
7	99.897998
8	99.898010
9	99.897998

From the above results we can see that, the identification value and theory value 100kg of the quality for the target are closer with the improvement of the data accuracy, but the accuracy of the identification results improved little, this shows the theoretical accuracy of the established model.

**Fig 7:**
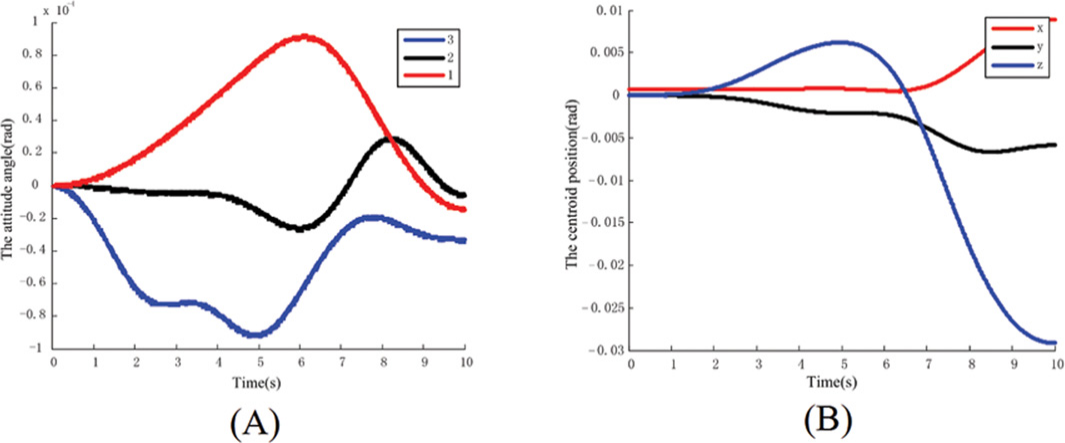
The absolute error of base in identified model. (A) Absolute error of base’s Euler angular. (B) Absolute error of base’s position.

Putting the identification results into the dynamics, the absolute error of the attitude angle (in accordance with the 3-2-1 rotation sequence) and the centroid position for the base are shown in the Fig 7.

Some information can be obtained by calculating the above data. When the identification results were inputted into the dynamics model of system, based on the same movement pattern of the arm, the maximum relative error of attitude angle for the base is 0.25%, the maximum relative error of the position for the base is 0.04%. Because the relative error of the pose and position for the base is very small, which can be ignored, it can be considered that the identification results are accurate.

The simulation results show that there are some errors for the parameters obtained by identification, but it doesn’t obviously have effect on the base attitude and kinematics, which can be ignored. This error is due to the lack of data precision. Setting the base initial centroid position as the original point of the inertial frame, the angular momentum is calculated and the result is not zero, but with a magnitude of 10^-2^m^2^kgs^-1^, which is caused by the truncation error of the selected data. Considering the error propagation effect, when there exists a certain error for joint angle and angular velocity, it will lead to the error of the motion parameters of each component for the system, so the system no longer meets the assumption that linear and angular momentum is constantly equal to zero, which is the reason for the identification error.

In terms of the parameter identification, compared with the measurement for the dynamic response characteristics, the computing time is shorter, which can be ignored. Because there are 10 inertial parameters in 3D space for the target, if employing the traditional identification methods, three times identification are required, which will consume much more time. Taking the numerical simulation model as an example, when using the traditional identification methods, the system response time is 30s while for the coordinated parameter identification, it is only 10s. Through optimizing the motion of the joint hinge for the manipulator, the system index can satisfy certain constraints during the identification, meanwhile, the system can become a specific configuration in the terminal state, which contributes to the next operation.

## Conclusion

In this paper, we study a parameter identification technique for the unknown target with of multi-joint about space precise operation of the non-cooperative target. The works contain: making review and summary of the existing parameters identification techniques for quality characteristic, and from the point of reliability and operability of the algorithm, we determine a parameter identification technique for the quality characteristic based on the conservation of linear and angular momentum; then the basic theory and algorithm of the coordinated identification were illustrated according to the principle of linear and angular momentum conservation, and the problems in calculation were analyzed and treated; at last we took the spacecraft with two 6-DOF manipulators capturing non-cooperative target as an example to perform the numerical simulation for the coordinated identification algorithm. The main conclusions obtained are as follows:

1. Only multi-hinge 3D motion data at two times is required theoretically to carry out one-time valid identification, and as long as the matrix is nonsingular, namely the data is effective, the identification results obtained at every time should be the same.
2. The identification technique has a high accuracy; and because the linear and angular momentum conservation has been substituted in derivation, the resulting algorithm requires less computation compared to those identification algorithms that must calculate the unknown system momentum.
